# The Thickness Effect of PSF Nanofibrous Mat on Fracture Toughness of Carbon/Epoxy Laminates

**DOI:** 10.3390/ma14133469

**Published:** 2021-06-22

**Authors:** Hamed Saghafi, Ali Nikbakht, Reza Mohammadi, Dimitrios Zarouchas

**Affiliations:** 1 Department of Mechanical Engineering, Tafresh University, Tafresh 3951879611, Iran; saghafi@tafreshu.ac.ir; 2 New Technologies Research Center (NTRC), Amirkabir University of Technology, Tehran 1591633311, Iran; 3 Department of Mechanical Engineering, Amirkabir University of Technology, Tehran 158754413, Iran; rezam69@aut.ac.ir; 4 Structural Integrity & Composites Group, Faculty of Aerospace Engineering, Delft University of Technology, 2629HS Delft, The Netherlands

**Keywords:** carbon/epoxy, PSF nanofibers, electrospinning, mode-I fracture toughness

## Abstract

The geometrical features of nanofibers, such as nanomat thickness and the diameter of nanofibers, have a significant influence on the toughening behavior of composite laminates. In this study, carbon/epoxy laminates were interleaved with polysulfone (PSF) nanofibrous mats and the effect of the PSF nanomat thickness on the fracture toughness was considered for the first time. For this goal, the nanofibers were first produced by the electrospinning method. Then, double cantilever beam (DCB) specimens were manufactured, and mode-I fracture tests were conducted. The results showed that enhancing the mat thickness could increase the fracture toughness considerably (to about 87% with the maximum thickness). The toughening mechanism was also considered by presenting a schematic picture. Micrographs were taken using a scanning electron microscope (SEM).

## 1. Introduction

Carbon fiber-reinforced polymers (CFRPs) are among the most common composite laminates used in different industries, especially in the automotive and aerospace industries. Despite their advantages, such as a high specific strength and elastic modulus, they can be easily damaged under fatigue, impact, or other loading [1,2,3,4]. The main damage modes of composite laminates are matrix cracking, fiber breakage, matrix/fiber deboning, and delamination. Up until now, many studies have focused on the latter (i.e., delamination) and have strived to remove, or at least decrease, the effect of delamination on the final failure mode of composite structures. Various techniques have been presented for this goal, with their advantages and disadvantages detailed in each study [5,6,7,8]. For instance, stitching is one of the most famous methods for increasing the delamination strength of laminates [9], but Yudhanto et al. [10] showed that it can also decrease the compression strength of laminates by up to 16%.

Using thermoplastics as additives in thermoset-based CFRPs is an attractive method for enhancing their fracture toughness [11,12,13,14]. These additives can be in the form of particles [15,16,17,18], film [19,20], or fibers [21,22,23,24,25,26], and range in size from nano- to micrometers. These types of materials have long been used for toughening laminates, except for nanofibers which were introduced in the last few years [27] and have not attracted researchers’ attention until recently [28]. In the last decade, many studies have been conducted on this topic, including a study on the effect of different polymers on increasing or decreasing fracture toughness, or the influence of the loading type on the effectiveness of nanofibers [29,30,31,32,33].

Up until now, the abilities of some polymeric nanofibers, such as polyvinylidene fluoride (PVDF) [34,35,36,37], nylon [38,39,40,41,42,43], and carbon nanofibers [44,45], have been considered for increasing the fracture toughness of composite laminates. However, the consideration of other factors such as the effects of nanofiber diameter, mat thickness, and nanofiber orientation is required to properly utilize nanofibers in the toughening of composite materials in real applications. For example, the effect of the mat thickness of nylon 66 and PVDF was considered by Brugo et al. [46] and Saghafi et al. [36,47]. They showed that increasing the mat thickness led to an enhancement of mode-I and mod-II fracture toughness. In two separate studies by Kheirkhah Barzoki [26,48], the effects of polyvinyl butyral (PVB) nanofiber diameter, orientation, and nanomat thickness on fracture toughness were considered, and it was found that there is an optimum value for the diameter and thickness of nanofibers.

As can be seen in these studies, considering the effect of mat thickness is important for gauging the toughening ability of nanofibers. Thus, in this study, the focus is on composite laminates toughened by polysulfone (PSF) nanofibers. Although there are some limited studies of this topic [49,50,51,52], no data have been published regarding the influence of PSF mat thickness on fracture toughness. The nanofibers used in this study were produced by the electrospinning method and were interleaved between composite layers. After conducting fracture tests, a scanning electron microscope was utilized for determining the toughening mechanism.

## 2. Production of Polysulfone Nanofiber by the Electrospinning Method

Electrospinning is one of the most common methods for producing nanofibers. Conductive collectors, high voltage sources, and syringe pumps with Teflon tubes are the most important components of an electrospinning machine [53]. In this research, polysulfone polymer (PSF) in the form of pellets was applied to produce nanofibers. To achieve this, Udel^®^ 3500 PSF was purchased from Solvay company (Milan, Italy) with the following properties: density, 1.24 g/cm^3^; melting point, 316–371 °C; tensile strength, 70.3 MPa (these data were obtained from Solvay’s website).

N,N-Dimethylacetamide (DMAc) and acetone were supplied as the solvent. The polymeric solution was prepared using 23% wt/v of polysulfone with solvent ratios of DMAc to acetone (90/10 *v/v*). The prepared solution was then transferred to the electrospinning machine and the following parameters were set to produce a uniform 3 ± 3 μm-nanofibrous mat: applied voltage, 22 kV; feed rate, 1.2 mL/h; and the distance between the needle tip and the collector, 12 cm.

## 3. Specimen Fabrication

**Figure 1:**
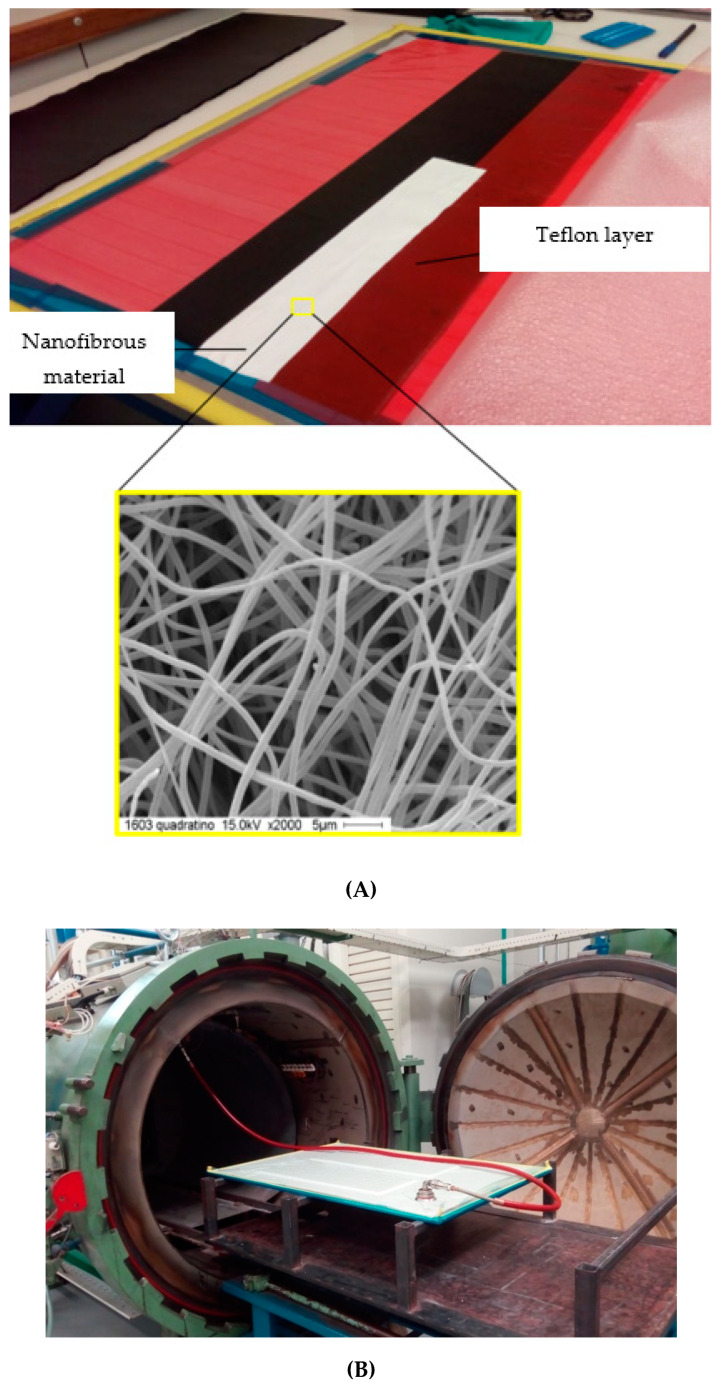
(**A**) The laminating process and (**B**) the curing process in the autoclave.

To produce the required samples, 24 layers of AS4/8552 carbon/epoxy prepreg (purchased from Hexcel company, Vise, Belgium) were stacked on each other. In the first step, 12 layers of prepreg were laminated, and a thin refractory Teflon layer was applied (as an initial crack). An additional 1, 2, or 3 mats of PSF nanofibers (used as a toughening agent) were implanted between these layers. In the second step, another 12 layers were placed onto the initial layers to produce the final sample. The laminated plate was then vacuumed and placed in the autoclave for curing. The autoclave control system was set according to the datasheet provided for the 8552 epoxy [54]. The heating rate in the autoclave was set to 1 °C/min. Figure 1 shows the laminating and curing processes of the composite plates in the autoclave. The cured laminates were cut into the final test specimens with a grinding machine.

## 4. Mode-I Quasi-Static Test

**Figure 2:**
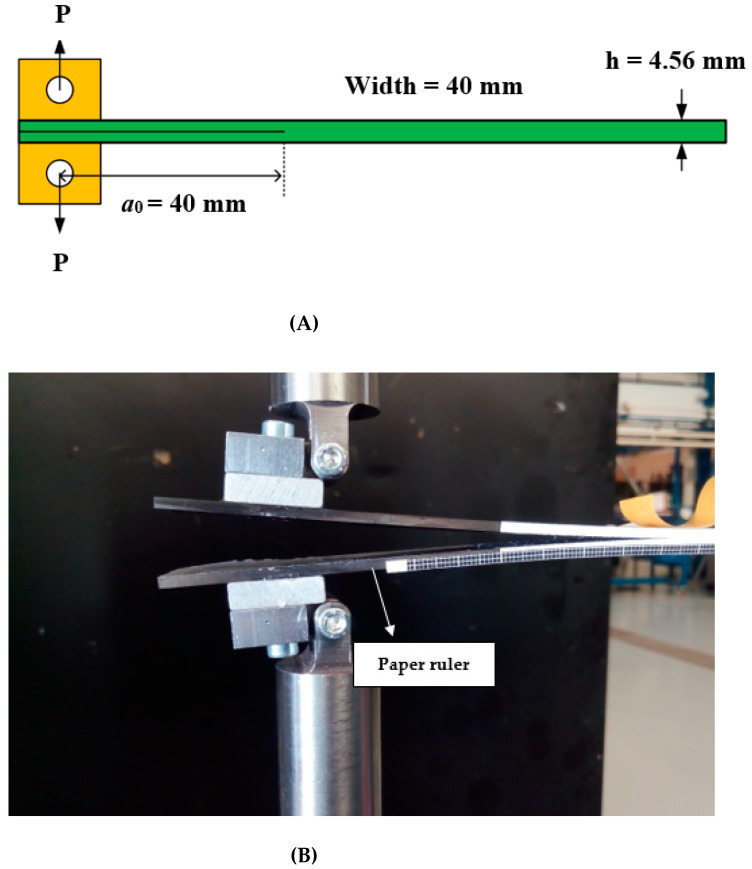
(**A**) DCB specimen characterizations; (**B**) mode-I experimental test setup.

In this research, double cantilever beam (DCB) specimens were used to determine the mode-I interlaminar fracture toughness. The test method and specimen size were followed according to the ASTM D5528 standard [55] where the width and thickness of the specimens were measured at three different points, and then, the average value of these dimensions was reported (Figure 2A).

All the tests were conducted using an MTS-10 kN universal machine at a displacement control condition (rate of 1 mm/min). The machine was carefully calibrated before conducting the tests. The relative error of the load cell and the displacement of this machine were 0.86% and 1%, respectively.

Figure 2 presents the characterization of the DCB specimen and test setup. Two aluminum loading blocks, 25 mm width by 20 mm length with 6 mm thickness, were adhesively bonded to the specimen for the load introduction.

A paper ruler with a grid size of 1 mm × 1 mm was attached to the edge of the specimen for the delamination length measurements and for investigating the variation of the fracture toughness during crack propagation (plotting R-curve). A paper ruler was attached to the samples for investigating the variation of the fracture toughness during crack propagation and plotting the R-curve. In order to investigate the test’s repeatability, three specimens were considered for each type. The sample specifications and their abbreviations are listed in Table 1.

## 5. Results and Discussion

**Figure 3:**
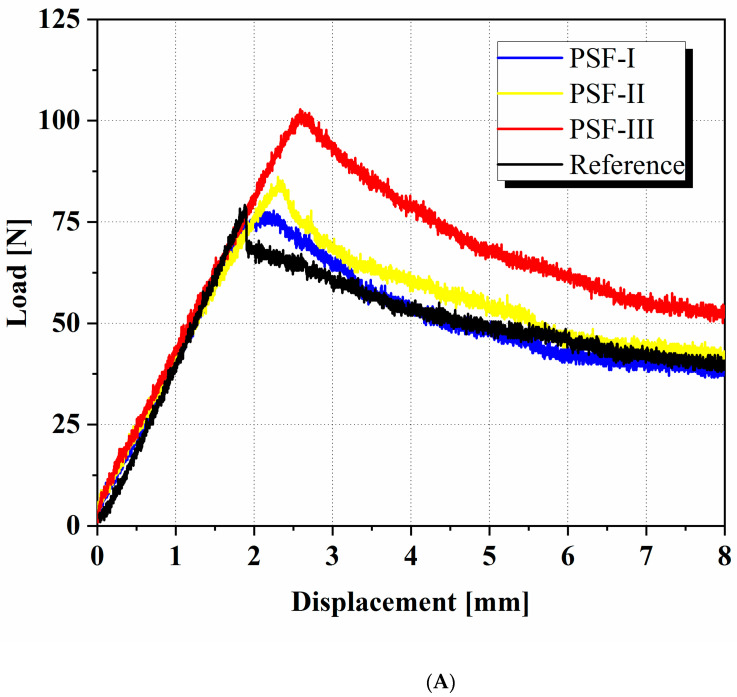

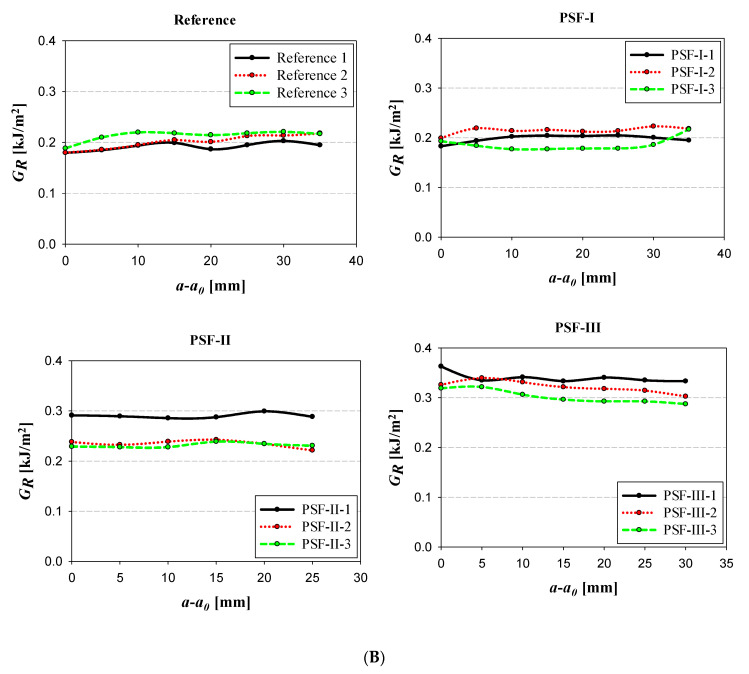
(**A**) The load–displacement curves for the reference and PSF-modified specimens, and (**B**) the R-curve for all the specimens.

The load versus load point displacement curves are presented in Figure 3A for all the types of specimens. For improved readability, only one curve of each type is plotted. At a first glance, the slope of the curves is nearly identical for all the samples; therefore, the addition of nanofibers did not affect the slope. This fact was also observed by other researchers who investigated other kinds of nanofibers [46]. Among the samples, PFS-III could stand the maximum load of 102.4 N. The value of this parameter was 79.2, 77.8, and 86.2 N for the reference, PSF-I, and PSF-II samples, respectively. An important point can be also observed in this figure; in the reference laminates, a force drop (about 15 N) occurred at the moment of initial fracture, while the force decreased slowly and continuously for the other modified samples. This occurrence proves the brittle fracture rate of the unmodified sample against the modified ones. This phenomenon occurs due to the nanofibers having introduced obstacles against the crack initiation, preventing the crack from achieving sudden propagation. The toughening mechanism in these samples is considered in the last section.

According to the ASTM-D5528 standard [55], Equation (1) was utilized for obtaining the mode-I interlaminar fracture toughness (*G_IC_*):

(1) $$G_{IC}=\frac{3p_{cr}\delta_{cr}}{2B(a_{0}+\Delta )}$$

where $p_{cr}$ is the critical force at which the crack starts to propagate, *δ_cr_* is the displacement corresponding to $p_{cr}$, *a_0_* is the initial crack length (40 mm), *B* is the specimen width (25mm), and ∆ is the crack length calculated according to the suggestion in ASTM D5528 [55]. 

Table 2 summarizes all the test results, and Figure 3B shows the R-curve for the reference and nanomodified laminates. As can be seen, the critical force in the case of PSF-I was less than the reference, but due to a larger *δ_cr_*, the average *G_IC_* increased by about 6%. Increasing one more layer of PSF between the laminate had a significant effect on the maximum load and fracture toughness and enhanced the *G_IC_* by about 41%. In the last modified sample (PSF-III), increasing the nanofibrous mat thickness led to an enhancement of the interlaminar fracture toughness (by about 87%). The interesting fact in this is the difference between the various polymeric nanofibers and how their thickness influences *G_IC_*. Brugo et al. [46] used 40 μm and 90 μm thickness mats in carbon/epoxy laminates. Their research showed that increasing the mat thickness increased the fracture toughness in woven laminates considerably, but its effect in unidirectional laminates was found to be negligible. The influence of the PVDF mat was also considered with two thicknesses of 30 μm and 60 μm by Saghafi et al. [47]. The outcomes showed that *G_IC_* increased by 42% and 98% by using the thin and thick membranes in comparison with the reference laminate, respectively. Unlike the above results, the effect of the thickness increase of the PVB nanofibers did not enhance the fracture toughness [56]. Kheirkhah Barzoki et al. [56] applied three different thicknesses of nanofibrous mats (25 µm, 45 µm, and 65 µm) between composite laminates. The thinnest mat had the highest rate of effectiveness.

The variation of the fracture toughness during the crack propagation is illustrated in Figure 3B. The PSF-I and the reference had similar responses during the tests. The interesting point in these tests was the behavior of PSF-II. As observed in Figure 3A, this case had a higher force in comparison with PSF-I and the reference, up to a displacement of about 5.5 mm. After this specific point, the force in all three samples was almost the same, although the fracture toughness of the PSF-II was more than the other two specimens, according to Figure 3B. The reason for this is that, with an equal amount of displacement, the crack length in the PSF-II is less in comparison with the other two samples. The PSF-III consistently had a higher fracture toughness in comparison with the other samples, although the maximum difference was observed at the beginning of the fracture process.

## 6. SEM Analysis

**Figure 4:**
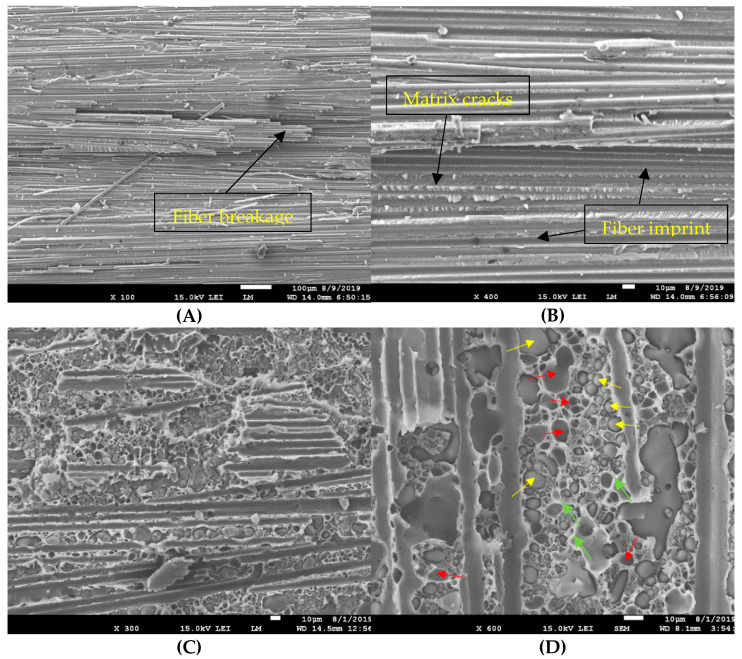
Fracture surfaces of (**A**,**B**) the reference and (**C**,**D**) the nanomodified laminates.

The SEM micrographs of the fractured surfaces are shown in Figure 4. In the reference test, the fracture of the epoxy is completely visible. The dominant failure modes in this case were matrix cracking (in the form of hackles), fiber breakage, and fiber/matrix debonding (in which the fiber imprints for the latest test can be observed on the fracture surface in Figure 4B). For the PSF-modified laminates (Figure 4C,D), phase separation occurred during the curing of the epoxy, which led to a sea–island structure in the PSF/epoxy. This means that the PSF spherical particles (shown by yellow arrows) were in the continuous epoxy phase. Since the PSF is a thermoplastic polymer and its viscosity is high, the produced spheres did not place far from the original nanofiber direction and position [57]. The PSF enhanced the fracture toughness for the following reasons: 1. A large number of cavities were associated with the pull-out of PSF particles (shown by red arrows). These particles made a bridge between the adjacent layers, departing from one layer and transferring to the other by absorbing energy. 2. The PSF microspheres caused crack deflection during delamination. As PSF is much tougher than epoxy, the cracks deviated from their original routes instead of breaking the PSF particles. This mechanism absorbed more energy, which led to an increase in *G_IC_*. 3. The local plastic deformation of the epoxy can be observed next to the PSF particles (shown by green arrows in Figure 4D) which can also absorb energy. Figure 4C presents the toughening mechanism in a higher magnification. Highly deformed epoxy is visible in the fractured surface of the laminate.

**Figure 5:**
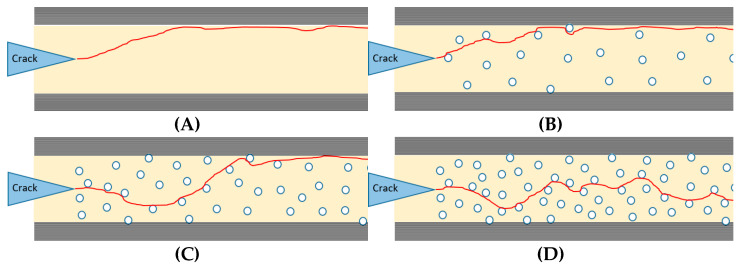
Crack propagation in the (**A**) reference, (**B**) PSF-I, (**C**) PSF-II, and (**D**) PSF-III.

The effect of the nanofibrous mat thickness on the fracture toughness is schematically illustrated in Figure 5. When the thickness was enhanced, the number of spherical particles increased. In this way, the number of crack deviations also increased. This meant that the crack traveled on a longer path. Both these parameters increased the required energy needed for crack propagation. Enhancing the thickness of the mat can also increase the possibility of other toughening mechanisms occurring, such as bridging caused by particles.

## 7. Conclusions

One of the most effective methods for toughening composite laminates is applying nanofibrous mats between composite layers. As has been proven, polysulfone (PSF) nanofibers are among the most suitable choices for this aim. The influence of mat thickness (30 µm, 60 µm, and 90 µm) on mode-I fracture toughness was considered in this study. By conducting tests and investigating the fractured surfaces by SEM, the following conclusions were drawn:

1. The influence of 30 µm nanomat is negligible on fracture toughness;
2. Increasing the mat thickness to 60 µm and 90 µm causes a 41% and 87% increase in the *G_IC_*, respectively;
3. The fracture toughness of laminates is enhanced through the following mechanisms: bridging between layers, crack deviation, and the local plastic deformation of epoxy;
4. Increasing the mat thickness leads to an increase in the number of PSF particles between composite layers. Thus, the possibility of the aforementioned toughening mechanisms occurring is also increased.

## Figures and Tables

**Table 1:** The specification of samples and their abbreviations.

Sample Specifications	Abbreviation	Nanofiber Thickness (µm)
Specimens without toughener(reference)	Ref	0
Specimens with 1 layer of polysulfone nanofibers	PSF-I	30 ± 2
Specimens with 2 layers of polysulfone nanofibers	PSF-II	60 ± 3
Specimens with 3 layers of polysulfone nanofibers	PSF-III	90 ± 5

**Table 2:** Test parameters and fracture toughness values for the reference and PSF-modified samples.

Specimen Code	*δ1_cr_* (mm)	*P_cr_* (N)	*Δ* (mm)	*G_IC_* (kJ/m^2^)	*δ_cr_* (Ave.)	*P_cr_* (Ave.)	*Δ* (Ave.)	*G_IC_* (Ave.)	Increase Percent(%)
Ref-1	1.87	84.8	8.36	0.19613	1.9 ± 0.037	79.52 ± 5.1	10.46 ± 2.57	0.179 ± 0.017	
Ref-2	1.88	79.2	9.69	0.17917					
Ref-3	1.94	74.6	13.33	0.16213					
PSF-I-1	2.02	75.1	9.61	0.18311	2.07 ± 0.045	75.68 ± 1.9	8.91 ± 1.16	0.191 ± 0.007	6
PSF-I-2	2.11	77.8	9.56	0.19816					
PSF-I-3	2.08	74.1	7.58	0.19395					
PSF-II-1	2.51	95.9	9.35	0.29181	2.39 ± 0.108	88.17 ± 6.9	9.88 0.739	0.253 ± 0.033	41
PSF-II-2	2.30	86.2	9.58	0.23896					
PSF-II-3	2.36	82.5	10.73	0.22971					
PSF-III-1	2.63	108.4	6.89	0.36335	2.63 ± 0.015	103.15 ± 4.9	8.22 ± 1.15	0.336 ± 0.023	87
PSF-III-2	2.61	102.4	8.93	0.32649					
PSF-III-3	2.64	98.7	8.85	0.31918					

## Data Availability

Data is available within the article.
